# Germinal Center B Cell-Like (GCB) and Activated B Cell-Like (ABC) Type of Diffuse Large B Cell Lymphoma (DLBCL): Analysis of Molecular Predictors, Signatures, Cell Cycle State and Patient Survival

**Published:** 2007-12-12

**Authors:** S. Blenk, J. Engelmann, M. Weniger, J. Schultz, M. Dittrich, A. Rosenwald, H.K. Müller-Hermelink, T. Müller, T. Dandekar

**Affiliations:** 1 Department of Bioinformatics, University of Würzburg, Biozentrum, Am Hubland D-97074 Universität Würzburg, Germany; 2 Institute for Pathology, Josef-Schneider-Str. 2, 97080 Würzburg, Germany

**Keywords:** regulation, gene expression, cancer, immunity, prognosis

## Abstract

Aiming to find key genes and events, we analyze a large data set on diffuse large B-cell lymphoma (DLBCL) gene-expression (248 patients, 12196 spots). Applying the *loess* normalization method on these raw data yields improved survival predictions, in particular for the clinical important group of patients with medium survival time. Furthermore, we identify a simplified prognosis predictor, which stratifies different risk groups similarly well as complex signatures.

We identify specific, activated B cell-like (ABC) and germinal center B cell-like (GCB) distinguishing genes. These include early (e.g. CDKN3) and late (e.g. CDKN2C) cell cycle genes.

Independently from previous classification by marker genes we confirm a clear binary class distinction between the ABC and GCB subgroups. An earlier suggested third entity is not supported. A key regulatory network, distinguishing marked over-expression in ABC from that in GCB, is built by: ASB13, BCL2, BCL6, BCL7A, CCND2, COL3A1, CTGF, FN1, FOXP1, IGHM, IRF4, LMO2, LRMP, MAPK10, MME, MYBL1, NEIL1 and SH3BP5. It predicts and supports the aggressive behaviour of the ABC subgroup. These results help to understand target interactions, improve subgroup diagnosis, risk prognosis as well as therapy in the ABC and GCB DLBCL subgroups.

## Introduction

Diffuse large B-cell lymphomas (DLBCL) are the most frequent B cell Non-Hodgkin’s lymphomas. Diagnosis relies at present on morphological, immune-phenotypic and laboratory parameters. Clinically, the International Prognostic Index (IPI; age, tumor stage, serum lactate dehydrogenase concentration, performance status, and the number of extranodal disease sites) (The International NHL Prognostic Factors Project, 1993) is often used to predict outcome in DLBCL. On the molecular level, gene expression signatures have been defined that predict outcome in DLBCL independent of the IPI (Rosenwald et al. 2002).

Alizadeh et al. (2000) investigated the gene expression patterns of “diffuse large DLBCL, follicular lymphoma and chronic lymphatic leukemia. They identified two novel distinct types of the DLBCL by gene expression profiling. The “activated B cell-like DLBCL”(ABC) group has a lower overall survival rate than the “germinal centre B cell-like DLBCL” (GCB) group. Von Heydebreck et al. (2001) applied their class discovery method ISIS on a subset of 62 samples and 4026 clones of the data by Alizadeh et al. (2000) and confirmed for these data the two entities ABC and GCB. The survival analysis of Rosenwald et al. (2002), assigned several genes to gene expression signatures and based on this an outcome predictor of survival. The constituents are the “Germinal-center B-cell signature”, “MHC class II signature”, “Lymph-node signature”, “Proliferation signature” and the gene “BMP6”. The predictor has a greater prognostic power in classifying patients into risk groups than the IPI (The International Non-Hodgkin’s Lymphoma Prognostic Factors Project 1993). Starting with 36 well known DLBCL prognosis genes from the literature, Lossos et al. (2004) found a six gene based outcome predictor and applied it to the data sets of Alizadeh et al. (2000) and Rosenwald et al. (2002). The latter one is an ongoing study and thus an extension and revision of the old data from Rosenwald et al. (2002) was possible for us (see Material and Methods).

In this study we investigate first the robustness of the data (Rosenwald et al. 2002) with respect to advanced and more appropriate normalization methods. For that, “loess” and “scale” are performed on the data set, as we are aware, for the first time and the results are discussed. Next, unbiased statistical classification analysis confirms for this enlarged data set the classical subgroups ABC DLBCL and GCB DLBCL independent from hierarchical clustering. Furthermore it supports those subgroups being homogeneous entities in the data.

Our analysis includes the expression values for the above 36 DLBCL prognosis genes and we apply more adequate tools from the Bioconductor library (Gentleman et al. 2004) to derive better predictors than e.g. the six-spot predictor found by (Lossos et al. 2004). Moreover, we identify and demonstrate that expression of early and late cell cycle genes distinguishes well the pathological entities ABC and GCB DLBCL.

Finally, we show that the most significant gene expression differences found including cell cycle genes, classical marker genes and all best separating genes are integrated into a compact key regulatory network with clear expression differences between both diffuse large B-cell-lymphoma subgroups. This finding is confirmed comparing the average distribution of genes on the Lymphochip and the connection distances between them in the human interactome as well as by confirming key gene expression differences found in our main data set from new analysis of further gene expression data by Shipp et al. 2002. A picture emerges where a central regulatory circuit tunes immune signatures, apoptotic and proliferation pathways in different ways between ABC and GCB DLBCL. The introduced methods can also be applied to other studies of gene expression analysis in cancer to establish improved prognosis predictors, identify regulatory circuits and for proper group classification.

## Materials and Methods

### Gene expression data and materials

Patient samples were obtained after informed consent and were treated anonymously during microarray analysis. DLBCL lymph-node biopsies were either snap frozen, frozen in OCT or disaggregated and frozen as a viable cell suspension. DLBCL gene expression was measured with cDNA arrays containing genes preferentially expressed in lymphoid cells or genes known or presumed to be part of cancer development or immune function (“Lymphochip” microarrays (Alizadeh et al. 1999)). Our array includes spots to measure individual exons of the same gene which may be expressed differently in both lymphoma subgroups.

### Microarray procedures

Fluorescent images of hybridized microarrays were obtained using a GenePix 4000 microarray scanner (Axon Instruments). Images were analysed with ScanAlyze (M. Eisen; http://www.microarrays.org/software), and fluorescence ratios (along with numerous quality control parameters; see ScanAlyze manual) were stored in a custom database. Single spots or areas of the array with obvious blemishes were flagged and excluded from subsequent analyses. Messenger RNA was extracted according to standard procedures (Sambrook and Russel, 2001) from tumor biopsy specimens of DLBCL patients. All cDNA microarray analyses were performed using poly-(A)+ mRNA (Fast Track, Invitrogen). For each hybridization, fluorescent cDNA probes were prepared from an experimental mRNA sample (Cy5-labelled) and a reference mRNA sample (Cy3-labelled) consisting of a pool of nine lymphoma cell lines (Raji, Jurkat, L428, OCI-Ly3, OCI-Ly8, OCI-Ly1, SUDHL5, SUDHL6 and WSU1). The use of a common reference cDNA probe allows the relative expression of each gene to be compared across all samples.

The original data generated by Rosenwald et al. (2002), in which the subgroups were defined by hierarchical clustering was provided to us by the authors. In our study we analyse an enlarged data set as follows: more patients (a total of 248 patients, each patient array included 12196 gene spots corresponding to 3717 genes), including a more recent classification. The outcome of this are 12.3% more ABC and 5.2% less GCB patients. 19 patients have been removed from the ABC and GCB groups. In detail, five ABC patients were removed from the earlier ABC classification, however, 14 other ones are now associated with it. From the earlier GCB group, 14 patients were assigned to other entities and 11 other patients were newly classified as GCB. Altogether, 25 patients were thus newly recruited into these two groups. Moreover, each spot is now analyzed in the new study individually. There was no pooling of data on datapoints (spots) as done in older analyses (Rosenwald et al. 2002). We further fully account for the changes in patients analysed (described above) by such an individual spot analysis. In summary this yielded about 3.3 times more data points per patient.

**Statistical analyses** were performed using the statistical software package R (R Development Core Team 2005) and Bioconductor (Gentleman et al. 2004). For normalization of gene expression data, methods such as vsn, loess and scaling methods were used. To detect differentially expressed genes, functions from the Bioconductor package “limma” were applied. Its special strength is the robust statistics based on linear models and a moderated t-test statistics including multiple testing correction methods (Smyth, 2005, pp 397–420; Smyth, 2004). Based on diagnostic plots we chose gene expression normalization using within-array and between-array normalization methods. The within-array normalization “loess” (Yang et al. 2001, pp 141–152; Yang et al. 2002) adjusts expression log-ratios in the way that they average to zero within each array to make genes on one array comparable to each other. We applied the “scale” method (Yang et al. 2001, pp 141–152; Yang et al. 2002; Smyth and Speed, 2003) for between-array normalization. It scales log-ratios to have the same median-absolute-deviation (MAD) across arrays. By this, log-ratios are normalized to show similar variance across a batch of arrays.

**Unbiased class discovery** was performed using the ISIS method (*i*dentifying *s*plits w*i*th clear *s*eparation; von Heydebreck et al. 2001). It searches for binary class distinctions in the gene expression levels in an unsupervised fashion. The diagonal linear discriminat score (DLD) quantifies for every found bipartition how strongly the two classes are separated. A maximum sample size of 150 patients for each ISIS run considered 3000 measurements and delivered 50 best separating genes.

**Cox regression hazard models** were done applying the R package “survival” (Andersen et Gill 1982; Therneau et al. 1990), to calculate the influence of gene expression values on the survival time and Kaplan Meier estimates. The outcome predictor score is calculated with the coefficients of the Cox model and the gene expression values.

**Supervised class analyses** were performed using “Prediction Analysis of Microarrays” (PAM) (Tibshirani et al. 2002). PAM performs a nearest shrunken centroid method to identify a subset of genes that best characterizes samples as ABC or GCB DLBCL. It computes a standardized centroid for each class and shrinks the prototypes for a given classification error threshold. In the resulting list the obtained optimal (for the given error) shrunken centroid identifier is followed by the number of genes it contains. The chosen classifier is validated by ten-fold cross-validation.

Smaller gene sets typically show larger error rates. However, if almost equally good performing classifiers existed, we parsimoniously chose the one containing the smallest number of genes. The proposed best gene set used for our analysis had 31 spots (labelled by an ‘x’ character in Fig. 2).

**Protein association networks** were identified by the STRING database, version 6.3 (von Mering et al. 2005), of known and predicted proteinprotein interactions. It combines information from genomic context, experiments, other databases, co-expression and text-mining. Homology predictions transfer and extend these data further. We used the STRING database with a Bayesian confidence level of 0.400 (medium confidence) and a custom limit of 0 (only direct interactions of proteins are considered).

## Results

### Improving prognosis prediction and separation of DLBCL subtypes

#### Statistical validation of the DLBCL subgroups ABC DLBCL and GCB DLBCL

**Figure 1:**
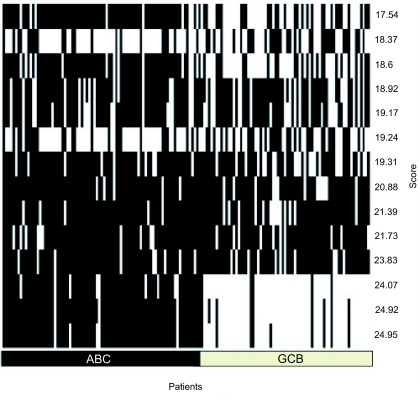
**DLBCL splits into sub-groups independent of signatures.** Optimal bipartitions of patients are calculated by ISIS based on optimal bipartition subsets of genes (50). Every column of the x-axis represents a patient. On the bottom, the DLBCL-type of the patient is labelled. On the y-axis every row shows the bipartitions ranked in increasing score of separation quality. The three best bipartitions show a very consistent and clear signal separating the ABC- from the GCB-patients. The unsupervised method ISIS reveals the ABC-GCB classification independent of proliferation signatures. No evidence for a previously suggested third group “Type 3” was found. Only a few patients are falsely assigned if compared to the DLBCL gene signature assignment.

Both subgroups were originally introduced on the basis of gene expression profiling. There has been some suggestion that certain diffuse large B-cell lymphomas form a third group (Hans et al. 2004). Furthermore, it was interesting to see whether this classification is also valid for this data set by an unsupervised classification method. To decide independently of any pre-clustering of specific marker genes whether there are two, three or even more lymphoma subgroups and whether they overlap with groups according to other group definitions (e.g. pathology). ISIS (see Materials and Methods) systematically investigates unsupervised all possible bipartitions of the gene expression data (excluding mediastinal lymphomas; see Materials and Methods) without prior knowledge of marker genes or signature pre-classification (Fig. 1). Nevertheless the bipartitions with the three highest separation scores support and identify the two pathological entities ABC and GCB. Distinct subgroups (splits) within the ABC or GCB entities are not validated by ISIS. In particular, no appropriate bipartition could be observed using previously putatively classified Type 3 patients and the ABC or GCB samples (data not shown). The precise separation into exactly these two subgroups is thus well supported even by an unbiased statistical method independent of predefined expression signatures.

#### Survival prognosis detection on the updated data and after advanced normalization

The signatures by Rosenwald et al. (2002) are independent from the clinical IPI score (see Introduction) and useful predictors within the low, medium and high IPI risk groups on their data set (Rosenwald et al. 2002). We now tested the performance of advanced normalization methods, namely the methods “loess” (Yang et al. 2001; Yang et al. 2002) and “scale” (Smyth and Speed, 2003; Yang et al. 2001; Yang et al. 2002) on our data set. The IPI score is considered here only as an independent and established clinical prognosis marker. On a normalized data set of 240 patients and considering all individual spots we utilised Kaplan Meier plots (Fig. S1) and reveal the good performance of the gene expression profiles (Rosenwald et al. 2002) also for this data set using the improved normalization procedure. The low risk IPI group in the renormalized data is not as well separated between the best and worst quartile as in Rosenwald et al. (2002). The separation of the high risk group is virtually unchanged. However, in the medium risk group a better separation was achieved by the renormalization and single spot analysis of the enlarged patient data. For the medium risk patients a better separation into high and low risk is particularly important for prognosis prediction. This method including the advanced normalization can also be applied to any other microarray data set.

#### An improved six-spot predictor for survival prognosis comparing multi- and univariate analysis

**Figure 2:**
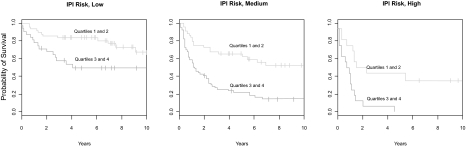
**Prognosis prediction applying a molecular predictor of 6 gene spots after improved normalization.** Kaplan-Meier plots show large differences in the survival rate for all risk groups. They are estimated by a Cox-Regression Hazard model of the genes listed in Table 1. Normalization was improved applying the “loess” method. x-axis: time (years); y-axis: probability of survival, predicted for the risk groups “low”, “medium” and “high”.

**Figure 3:**
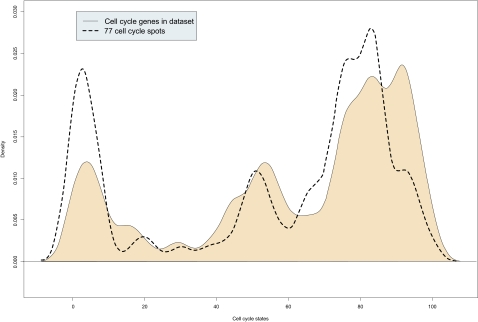
**Early and late cell cycle genes are overrepresented in the best separating cell cycle gene set.** The density plot compares the distribution of different cell cycle gene sets. x-axis: cell cycle states (from 0 to 99; complete cell cycle). y-axis: relative frequencies. Black line: density of all mapped cell cycle genes of de Lichtenberg et al (de Lichtenberg et al. 2005) in the data set. The area under this line is coloured for easier comparison. Blue line: Optimal separating subset of cell cycle genes (77 spots). Two peaks in the early and late cell cycle states show cell cycle gene expression differences between the subgroups ABC and GCB.

The immune signature requires the measurement of gene expression for many genes. We investigated whether a combination of array spots achieves similar good classification. Multivariate analysis (4 spots results in Table S1 and Table S2, they include immune genes) was computationally prohibitive for more than 4 spots. However, by univariate analysis we could systematically test the capability of gene expression values from individual spots to separate patients with good or bad prognosis in Kaplan-Meier plots. We considered for all three IPI classes the separation of best patient quartile with good prognosis from the worst patient quartile with poor prognosis. Using all genes and the 160 patients from the training-set we identified the spots predicting outcome best. Together, in a multivariate model, they form a predictor separating best and worst quartiles for all three IPI categories including the 80 patients from the validation-set. The five-spot-predictor considers different splicing forms in HLA-DRB5. Five spots (HLA-DPa, Brca, HLA-DQa, and two clones of HLA-DRB5; details in Suppl. Material) are about equal to the six gene predictor of Lossos et al. (2004). However, six genes and spots (HLA-DPa, HLA-DQa, HLA-DRb5, SEPT1, EIF2S2 and IDH3A genes, Fig. 2) show even an improvement for this classification task. The separation of the best and worst quartiles in the three IPI classes is comparable (Fig. 3) to the prediction success of the complete signature of Rosenwald et al. (2002) and classifies different patient quartiles better than the set proposed by Lossos et al. (2004; using LMO2, BCL6, FN1, CCND2, SCYA3 and BCL2 for overall survival in DLBCL). Our predictor is delivered by bioinformatical analysis of gene expression measurements, whereas Lossos et al. used real time PCR. However, our method can also be applied to real time PCR data.

Moreover, we tested the influence of the high correlation between the genes HLA-DPa, HLA-DQa and HLA-DRB5 on the quality of the predictor. The survival prediction with predictors of non correlated genes from the univariate analysis yields no improvement in the results (data not shown).

#### Genes best distinguishing DLBCL subgroups

Nearest shrunken centroid analysis using the R-package PAM (“Prediction Analysis of Microarrays”) identifies best separating genes for the two subgroups (ABC and GCB DLBCL) with smallest cross-validation error (Fig. S2). Gene numbers of classifiers are plotted versus the resulting error rates. The optimal classifier (Table S3) requires only 18 genes (31 spots) with an overall cross validation error of 6.2% (5 out of 82 ABC DLBCL samples were falsely predicted as GCB (6.1%); 7 out of 112 GCB DLBCL as ABC (6.25%)). Larger gene sets show similar error rates (see Materials and Methods), smaller gene sets result in inferior classification (Fig. S2). GCB DLBCL is correctly predicted even with fewer genes, however, the error for ABC DLBCL samples increases strongly (Fig. S2 lower plot). For clinical application both entities have to be well separated.

### Functional relationship of the genes differently expressed in ABC and GCB

#### Classical lymphoma gene-markers compared to the identified best separating genes

We tested whether 35 classical lymphoma genes (listed in Table S4; as described in Monti et al. 2005; Lee et al. 2003; Willis et al. 1999; Polo et al. 2004; Rosenwald et al. 2002) separate well the two major subtypes of DLBCL. Three metabolic enzyme genes for LDH (IPI score prognosis marker), IDH and PDH were added. Altogether these 38 genes correspond to 180 spots. PAM analysis identified a set of 9 well classfying genes (21 spots) (Table S5 and S6), with an overall error rate of 14% (10% training set; 15% for the validation group). However, the classical genes require more spots and their separation is not as good as the optimal prediction set above (Fig. S2). After this we merged these classical lymphoma marker genes with the best separating gene set found above for classification. We found, however, that here the best separating genes achieve all top ranks in this task (Table S7). Only mitogen-activated protein kinase 10 (MAPK10), the best classical lymphoma marker, reaches top ranks. BCL6 as the next best classical marker reaches only rank 31. Below we show that classical lymphoma genes are close to but not identical to the central regulatory network and genes best separating GCB and ABC DLBCL.

#### Cell cycle genes are differently expressed in ABC and GCB

Cell cycle is critical for cancer cell proliferation and we next investigated by PAM analysis (see Material and Methods) whether the functional group of cell cycle genes alone could separate the two B-cell lymphoma groups. We identified 473 spots, which correspond and are homologous to the cell cycle genes found by de Lichtenberg et al. (de Lichtenberg et al. 2005). These genes are annotated according to expression in the cell cycle state (100 steps between 0 and 99 for a full cell cycle).

The separation between the lymphoma subgroups improves as more genes are used. 77 cell cycle genes (Table S8, Table S9; error rate of 15.4%) yield low error rates using a medium sized gene set (classification optimum, see materials and methods). These include genes such as Butyrophilin-like protein 9 (BTNL9), early B-cell factor (EBF), TSC22 domain family member 1, Cyclin-G2 (CCNG2), Interleukin-6 (IL6), immediate early response protein 5 (IER5) and further homologues of typical cell cycle stage-specific genes (de Lichtenberg et al. 2005) such as TIMP metallopeptidase inhibitor 1(TIMP1) and v-maf musculoaponeurotic fibrosarcoma oncogene homolog (MAF), which mainly reflect the late cell cycle states. Figure 3 compares the complete cell cycle genes in our data set with the subset of 77 genes in a density plot. The black line indicates all cell cycle states of the whole chip and the blue line the subset of 77 genes. The densities of these gene sets clearly differ in the early (steps 0–18) and in the late steps (75–85) of cell cycle (p = 6.65·10^−10^; Wilcoxon one sided test).

Cell cycle spots, which show the biggest difference in gene expression values between ABC and GCB DLBCL, are in the late steps 72, 80, 84 and 85 (Fig. S3; M/A plot, ie,middle intensity of the genes against difference in expression of both lymphoma subgroups). Moreover, these cell cycle states form a compact cluster in the plot. This data indicate a clear difference in cell cycle states regarding the two DLBCL subgroups.

#### Cell cycle genes, classical lymphoma genes and best separating genes form a compact network important for DLBCL subtype distinction between ABC and GCB

Are the genes differentially expressed in ABC and GCB DLBCL specially connected, and in particular, if so, how do their respective gene products interact with each other? To analyze this systematically, different large scale protein interaction databases were investigated such as the hand curated HPRD database (Peri et al. 2003). The large proteinprotein interaction database STRING (von Mering et al. 2005) allowed us to establish an interaction network (Fig. S4, Fig. S5). Note that this analysis focuses on the clearly differentially expressed genes in ABC and GCB (Table S7). Classical lymphoma gene markers (dark grey boxes) as listed in Table S5 combine and interact with the compact cluster of the most powerful differentiating genes (white boxes) for the whole data set (Table S3) as delivered by PAM. The connections are mainly found by text-mining; however, the two interactions between BCL6—IRF4 and between SH3BP5—MAPK10 are available from the HPRD data set (experimental/biochemical data) as a direct physical interaction (blue). The different article sources re-examine the interaction predictions for different cancer entities: “DLBCL”, “no cancer disease” and “other cancer”. Note that these categories support the interactions from three different view points (Fig. S5). We find that 11 of the 18 best separating genes and 8 of the 9 separating classical lymphoma genes are members of this dense interaction network. This is supported by the interaction data, the HPRD database and various specific interaction evidence types collated by the STRING database.

The remaining 8 genes, 7 from the first mentioned set and 1 from the latter one, are not part of the databases. Cyclin D2 (CCND2) occurs in both subsets and we obtain a protein association network of 18 nodes. Regarding network regulation the underlined genes are higher expressed in ABC, all others are higher expressed in GCB subtype: ASB13, BCL2, BCL6, BCL7A, CCND2, COL3A1, CTGF, FN1, FOXP1, IGHM, IRF4, LMO2, LRMP, MAPK10, MME, MYBL1, NEIL1 and SH3BP5 (Table S10). The characteristics of the network are described in Table 2: Protein functions involved in the network include stimulation of proliferation, block of proliferation, apoptosis, differentiation and immune cell specific functions. Both DLBCL subgroups show clear differences in these specific pathways and sub-networks. Furthermore, the large collection of protein associations from the STRING database shows that all these different proteins separating the two subgroups are connected by first order interactions. As a control for this finding of a compact regulatory network separating both entities regarding gene expression, we tested that all Lymphochip genes are equally distributed with regard to the human interactome and not pre-clustered (Fig. S6). Moreover, the characteristic path length for randomly picked genes from the Lymphochip is 3.985 (Fig. S7) and clearly longer than the direct interactions (path lengths one or two) found for the differentially regulated network (Fig. S4).

Moreover, 5 of the 8 cell cycle genes, identified in Figure S3 above, to be regulated differently are directly interacting with this regulatory network (Fig. S5). The genes with a significantly higher expression in the ABC group are marked by a red rectangle, whereas green ellipses mark higher expression in GCB. These differences are an interesting pointer for a more specific anti-cancer treatment.

#### Gene functions for well separating genes

The shorter survival of patients with ABC DLBCL is connected to pathways expressed differently from GCB DLBCL; thus the well known BCL2, as a central apoptosis blocker is higher expressed and allows cancer cell survival in ABC DLBCL. BCL6, a transcriptional repressor important for B-cell differentiation, is down-regulated in ABC DLBCL. Altogether, apoptosis genes are lower expressed in the ABC DLBCL subtype.

Furthermore, the low gene expression values of the gene MME, a proliferation blocker, CCND2 and BCL7A, both genes which promote proliferation, and high values of SH3BP5 in the ABC DLBCL patients stimulate proliferation.

Both the immune cell specific genes IGHM and IRF4 are higher expressed in ABC DLBCL; however, all genes which are associated with differentiation are down-regulated.

In conclusion, this network indicates down-regulation of apoptosis and differentiation for the ABC DLBCL patients whereas the proliferation and immune cell stimulating genes are up-regulated.

From the cell cycle genes which are connected to the network, IL6 and IER5 show higher values in the ABC group whereas BTNL9 and CCNG2 show an up-regulation in the GCB group. For the latter it is known that CCNG2 and IL6 block the proliferation.

In order to further validate the found gene expression differences, we show that several of these are found again after analyzing further data from Shipp et al. (Shipp et al. 2002; Wright et al. 2003; Table S12).

Do the clear gene expression differences between both subgroups reflect only differences in B-cell specific regulation? In order to gain a first impression regarding T-cell regulatory pathways from our data we tested whether notch genes, trans-membrane receptors important in T cell differentiation and repressed in many cancers (Reizis and Leder, 2002), regulate differently the target genes in the two groups. Target genes are regulated by GY-box-, Brd-box-, and K-box-class microRNAs in the 3′-UTRs e.g. in Drosophila (Lai et al. 2005). We mapped all genes of the Lymphochip to the transcripts annotated in ensembl. We screened these and found candidate notch target genes, whose transcripts bear the mentioned target sequences. All three boxes were found in the genes given in supplementary Table S11. From these transcripts the “Deoxycytidine kinase” gene (ENSG00000156136, DCK) and the “Translocation associated membrane protein 2” (ENSG00000065308, TRAM2) show clear gene expression differences between the ABC and GCB subgroups.

## Discussion

### Marker genes for DLBCL subtypes

This study improves marker gene detection for prognosis and subtype diagnosis of diffuse large B-cell lymphomas (DLBCL) applying a wide range of methods useful also for other gene expression measurements in cancer. A special patient group are primary mediastinal B-cell lymphomas. Patients recognized with this disease (6 cases) were excluded from the data set and hence are neither visible nor contained in the further analysis. This is in accordance with previous studies (Rosenwald et al. 2002) and other data sets (Alizadeh et al. 2000; Shipp et al. 2002; Wright et al. 2003).

The classification of all other diffuse large B-cell lymphoma into two pathological entities has been established by marker genes and their expression (Alizadeh et al. 2000). A third entity has been discussed (Hans et al. 2004) but was disputed again in the light of recent data. Our statistical analysis by ISIS method (von Heydebreck et al. 2001) provides an independent method and validates and supports only these two subgroups. In addition to previous work (Rosenwald et al. 2002), ISIS analysis here clearly indicates for a large data set the bipartition of all patient data into the two subgroups ABC and GCB through an unbiased and independent statistical method. An adequate normalization of the gene expression intensities applying the loess method (Yang et al. 2001; Yang et al. 2002) allowed a better separation for best and worse outcome quartiles of survival, in particular for patients with medium IPI score where a better separation is important for accurate prognosis. We found a simplified (6 instead of 17 gene spots) survival predictor useful for clinical monitoring e.g. applying RT-PCR (Lossos et al. 2003). Multivariate analysis showed that a four-spot predictor does not perform well. However, univariate analysis found a six spot prognosis predictor which is superior to a previous six-spot predictor (Lossos et al. 2004) and to an alternative five spot predictor, in particular regarding high risk patients.

### Integrated picture of all gene regulation differences

Following this, the statistical analysis identified all genes which well distinguish the ABC and GCB DLBCL subgroups including differences in early and late cell cycle which could be exploited for a differential cytostatic therapy in the two subgroups.

We considered all the identified gene expression differences in order to obtain a detailed description of the differences between both DLBCL subgroups regarding regulation of the cellular network. We show that immune signatures, apoptotic and proliferation pathways are tuned in different ways between ABC and GCB DLBCL. A central circuit of genes is formed by genes that distinguish both lymphoma subgroups and are regulated differently. We also verified this for other data after completion of the first analysis. For the data in Shipp et al. (2002) and Wright et al. (2003) once again key genes from the central network shown in Figure S4 are confirmed as having a significant different regulation in this totally different data and patient set (Table S12). Classical lymphoma genes are either directly or indirectly interacting with it. Besides this central network other pathways are also implicated, we showed that two Notch pathway targets are specifically up-regulated. PAM has been shown previously to be a powerful method for gene selection (Tibshirani et al. 2002).

The different predictors shown in this study were the best predictors according to PAM curves and statistical analysis and gave clear improvements for prognosis prediction compared to previous studies (Rosenwald et al. 2002; Lossos et al. 2004) including a six spot predictor for clinical application. Furthermore, our results are based on experimental gene expression data on 248 patients and individual analysis of 12196 array spots whereas pooled data and fewer patients were used in older studies (Rosenwald et al. 2002; Lossos et al. 2004). Interesting marker genes were found in this study by different statistical methods (PAM, ISIS, LIMMA). Clearly, using other methods (e.g. support vector machines) different gene sets can be obtained. In our study, the ISIS method is applied for explorative analysis and unbiased classification without prior knowledge or gene signatures. It supports independently the two distinct B-cell lymphoma subgroups. The different gene sets were further validated against each other by including classical marker genes. Moreover, we validate in our study key marker genes we found by analysis of additional and further data (Shipp et al. 2002; Wright et al. 2003). A new perspective from this study is that genes found differently expressed in the two B-cell lymphoma types form a compact interaction network including cell cycle genes. This is obtained by another independent analysis method (protein-protein interaction database STRING). Furthermore, the delineated regulatory network adds biological data and data from large-scale interaction databases to show that the identified marker genes are in fact members of a closely interacting regulatory network, with molecular functions that mirror the differences in pathology of the two subgroups GCB and ABC DLBCL.

The identification of cell cycle genes expressed differently indicates here new possible targets for therapy. Differences between the ABC and GCB DLBCL subgroups are at the beginning and the end of the M-phase and the early part of the G1 phase. Inhibiting early cell cycle genes, overexpressed in ABC and adding known cytostatic drugs such as mitosis inhibitors and early G1 blocker may be particularly useful for ABC DLBCL patients. A more detailed therapy profile would take the further differences in regulation into account.

## Conclusion

The present analysis reveals through the use of an array of methods a detailed picture of molecular markers differentiating cancer subtypes. We apply it to GCB and ABC DLBCL for clinical use in determining prognosis and diagnosis. This included efficient six spot predictors for prognosis and clinical application. The entities ABC and GCB DLBCL have been confirmed by statistical analysis independent of gene expression signatures, a third entity could not be supported. The resulting genes with altered expression were found to form a tightly connected regulatory network including cell cycle genes, apoptosis and immune differentiation implicated in the aggressive behaviour of ABC DLBCL compared to the GCB DLBCL subtype.

## Supplemental Methods

To systematically identify spots which describe the outcome and cooperate well with each other in the Cox regression hazard model a multivariate analysis is desirable. However, this requires a huge search space of combinations to be tested. To reduce this we considered only four spot combinations of (i) the gene spots suggest by Rosenwald et al. (Rosenwald et al. 2002), (ii) the 36 important genes for diffuse large B-cell lymphoma chosen by Lossos et al. (Lossos et al. 2004) or (iii) the LDH-, IDH-, and PDH gene spots (the latter to better reflect IPI-scores). Cox Regression Hazard analysis was performed on all possible four tuples of these 153 indicator spots testing 160 patients (several days of calculation time on a LINUX cluster with 20 nodes of Pentium IV CPUs). Table S1 shows the gene content of the ten best multivariate four-spot-predictors (the next best combinations after removing these spots is found in Table S2). The best multivariate four-spot combination is compact and small, but neither as good as the five spot predictor in results nor as the signatures from Rosenwald et al. (Rosenwald et al 2002). The analysis further shows that there is a correlation with survival prediction for the clinical parameter LDH (Table S2), but the prediction based on this well known parameter (part of the IPI score) is even worse then the results shown in Table S2.
In contrast (see below), the new five-spot and six-spot predictors identified by univariate analysis will be useful heuristics for diagnosis and clinic, e.g. to identify risk quartiles and subgroups (Fig. S1).

Figure S1 **Kaplan Meier plots of the IPI groups.** The Kaplan Meier plots estimated by the molecular predictor of Rosenwald et al. (Rosenwald et al. 2002) applied on the new normalized gene expression data of the 240 diffuse large B-cell lymphoma patients. The plots show different groups according to their IPI risk and the training set as Training, Validation and all patients. The left column represents the training-group, the middle one the validation group and the right one all patients. The rows show the IPI risk groups. The first line shows low risk, the second one the medium risk and the last line the high risk patients. The x-axis is the time in years and the y-axis the probability of survival.

Figure S2. **PAM misclassification error of the ABC and GCB subgroups over all genes.** The upper plot shows the overall error while the lower one shows the subgroup specific errors. In both, the various thresholds on the lower x-axis correspond to different numbers of genes, labelled on the upper x-axis. The y-axis represents the error and ranges from 0 to 1. The good overall performance of PAM requires only few genes to decrease the error dramatically. The error rate decreases strongly between the thresholds of 6 and 5, which represent the amount of shrinkage. Hence we chose a threshold below 5 with the corresponding set of best separating genes (an optimal choice with few errors and a low number of genes). The performance for the single subgroups shows a big difference between ABC and GCB. Whereas GCB shows a good performance even with few genes, the prediction quality of ABC decreases dramatically in the case of ABC patients. This indicates a complex pattern of gene expression in ABC patients which is defined in more than 15 genes.

Figure S3. **Cell cycle genes with extreme expression differences shown by a MA-plot of normalized gene expression values.** The M values on the y-axis correspond to the gene expression difference between the ABC and GCB patient medians and the A values on the x-axis correspond to the average expression of all genes in both groups. The colored points represent the 77 cell cycle spots chosen by PAM analysis. The color scale ranges from yellow to red, whereas yellow is annotated to cellcycle state 0 and red to state 99. Additionally some cell cycle genes show more extreme A values(circle). They are labeled with their names and their cell cycle state. Remarkably, some genes associated with a late high cell cycle state cluster together regarding their gene expression values in both dimensions (ellipse). Again, late cell cycle states indicate a high difference in the M-value (difference in gene expression) between the two subgroups. A locally weighted regression smoothing line (lowess) shows that systematic and random variations are well controlled by the normalization procedure: Its shape fits almost perfectly the horizontal line.

Figure S4. **Regulatory network differently regulated in ABC and GCB B-cell lymphomas.** This figure shows the resulting network and interaction pattern with each other for the best separating genes applying data from the STRING meta-database of protein interactions. Classical lymphoma genes and best separating gene set form a tight network with the best separating genes in the centre. Shown are the strongly connected network members. They consist of (i) classical lymphoma marker genes (grey boxes), and (ii) the most powerful predictive genes in the PAM analysis (white boxes). Genes which show a significant higher expression in the ABC subgroup are marked by a red rectangle. They are associated to proliferation, block of proliferation, apoptosis, differentiation and specific for immune cells, as most of the remaining ones. Green ellipses mark higher expression in GCB. The almost fully connected gene network demonstrates that both classes of genes are well participating in the interaction network according to the STRING meta-database. Furthermore, the STRING analysis shows that almost all connections between both classes – the yellow colored edges - are based on literature (mainly Medline reports). Only the interaction of “interferon regulatory factor 4” (IRF4) and “B-cell CLL/lymphoma 6” (BCL6) is confirmed by large-scale interaction screen experiments.

Figure S5. **Regulatory network differently regulated in ABC and GCB B-cell lymphomas.** Functional protein association network using interactions predicted by the STRING database: the most powerful predictive genes in the PAM analysis (white boxes; see Figure 4S), classical textbook lymphoma genes (dark grey boxes), additional the cell cycle genes (light grey boxes; see Figure 3S: 5 of these 8 cell cycle genes are connected directly with the network. TIMP1 even connects the so far uninvolved classical lymphoma gene CTGF with the network. This indicates how well the cell cycle genes fit to the existing graph). The new connections are confirmed by text mining of PubMed abstracts(circles: DLBCL, diamonds: “no cancer disease”, empty square: “other cancer”); these different data complement each other. The genes with a significantly higher expression in the ABC group are marked by a red rectangle. Green ellipses mark higher expression in GCB. Black hexagons mark genes which have a very high average gene expression value in both entities and are an important part for the network.

Figure S6. **The Lymphochip genes in the human interactome.** This plot shows the human interactome as a protein interaction network. The proteins(circles) of the lymphochip are filled out. Interactions are drawn as a line. Characteristic path length and the longest path are 4.642 and 15, respectively.

Figure S7. **Histogram of the protein interaction distances.** The genes of the Lymphochip were mapped to the protein interaction graph in the human interactom. The histogram shows the occurring distances of these genes in the interactome. The longest distance is 11 whereas the characteristic path length is 3.985.

**Table S1.** Multivariate Cox regression hazard models.

**Nr**	**Multivariate Cox regression hazard model**			
1	HGAL	Germ-S	ACTa1	HLA-DRA
2	HGAL	CD54(2)	ACTa1	HLA-DRA
3	HGAL	CD54(2)	HLA-DRA(2)	ACTa1
4	HGAL	CD54(2)	HLA-DRA(3)	ACTa1
5	HGAL	ACTa1	HLA-DRA	CD54
6	HGAL	MHCIIDQa1	CD54(2)	ACTa1
7	HGAL	CD54(2)	MHCIIDRb	ACTa1
8	HGAL	Germ-S	MHCIIDRb	ACTa1
9	HGAL	Germ-S	HLA-DRA(2)	ACTa1
10	HGAL	Germ-S	HLA-DRA(3)	ACTa1

A heuristic search of multivariate Cox regression hazard models revealed this 10 best fitting models. All possible multivariate Cox regression hazard models of four 4 genes from 36 important genes for diffuse large B-cell lymphoma and the metabolic genes LDH, IDH and PDH were calculated and these ten gene sets fit best. Genes are abbreviated according to GenBank nomenclature.

**Table S2.** Next best multivariate Cox regression hazard models.

**Nr.**	**Multivariate Cox regression hazard model**			
1	CD10	IRF4	HLA-DRb5	LDH(2)
2	IRF4(2)	BCL7A	HLA-DRb5	LDH(2)
3	MYC	IRF4(2)	HLA-DRb5	LDH
4	MYC	IRF4(2)	HLA-DQa1	LDH
5	PLAU	IRF4	BCL7A	HLA-DRb5
6	IRF4	BCL7A	HLA-DRb5	LDH(2)
7	PLAU	IRF4(2)	BCL7A	HLA-DRb5
8	IRF4	BCL6	BCL7A	HLA-DRb5
9	CD10	IRF4(2)	HLA-DRb5	LDH(2)
10	MYC	IRF4(2)	HLA-DRb5	LDH(2)

If the genes appearing in Table S1 are removed, and the heuristic search of multivariate Cox regression hazard models is redone, these ten models are the next best fitting. The genes are represented by their GenBank abbreviation. The metabolic marker LDH from the IPI score occurs in the four best fitting models as well as in the the majority of the models.

**Table S3.** Genes which distinguish best between ABC and GCB according PAM analysis.

**Nr.**	**Gene**
1	MYBL1
2	*Centerin
3	FOXP1
4	LOC96597
5	SH3BP5
6	KIAA0864
7	IRF4
8	ASB13
9	*Similar to human endogenous retrovirus-4 Clone=417048
10	NEIL1
11	MME
12	IGHM
13	LMO2
14	LOC152137
15	KIAA1039
16	LRMP
17	FLJ123633
18	CCND2

From all twelve thousand spots from the lymphoma chip, the listed genes distinguish best between ABC and GCB according to PAM analysis. The best separating genes are written on the top.

**Table S4.** Classical lymphoma genes.

**Nr.**	**Gene**
1	BCL6
2	BRAF
3	ARAF1
4	RAF1
5	RAS
6	MEK
7	MAP
8	HLA-DPα
9	HLA-DQα
10	HLA-DRα
11	HLA-DRβ
12	α-Actinin
13	COL3A1
14	Connective-tissue growth factor
15	FN1
16	KIAA0233
17	PLAUR
18	E2IG3
19	NPM3
20	BMP6
21	CASP10
22	POU2AF1
23	CDKN2A
24	MYC
25	BCL2
26	FCGR2B
27	CyclinD1
28	NFKB2
29	PAX5
30	BCL10
31	CDK6
32	DDX6
33	BCL7A
34	CyclinD2
35	IL-10
36	LDH
37	IDH
38	PDH

Lymphoma associated genes were collected from literature and were also found in the data set. Furthermore we added the metabolic enzymes “lactate dehydrogenase”(LDH), “isocitrate dehydrogenase” (IDH) and “pyruvate dehydrogenase”(PDH). The latter are represented in the data by the genes PDHB, PDHA1, IDH3A, IDH3G, IDH3B, IDH1, IDH3B, IDH3A, LDHB and LDHA.

**Table S5.** Classical marker genes of lymphoma disease distinguish between ABC and GCB lymphoma subtype (PAM analysis; error rates for this gene set: TR:10% VAL:15.38%; F:CV:14%))

**Nr.**	**Gene**
1	FN1
2	BCL6
3	CTGF
4	BCL2
5	MAPK10
6	CCND2
7	COL3A1
8	KIAA0233
9	BCL7A

**Table S6.** Lymphochip spots of known lymphoma genes.

**SpotID**	**Gene Name**
19384	MAPK10
24787	CCND2
15914	MAPK10
24429	BCL6
28472	MAPK10
19268	BCL6
16858	CCND2
17646	BCL2
16789	BCL2
19361	COL3A1
26535	BCL6
28859	BCL2
24367	BCL2
17791	FN1
16016	FN1
16732	FN1
31398	FN1
19379	FN1
27499	KIAA0233
24415	BCL7A
29222	CTGF

180 spots, which are known to deal with lymphoma were tested to distinguish between ABC and GCB subtype by PAM analysis. Successful genes are given in descending order (gene set error rate:TR:10% VAL:15.38%; F:CV:14%)

**Table S7.** Combined classifier for lymphoma subtypes.

**SpotID**	**Gene Name**
24376	*Centerin
17496	MYBL1
28014	MYBL1
19326	IGHM
19254	MME
33991	FOXP1
19384	MAPK10
19375	FOXP1
16049	IGHM
26454	SH3BP5
22118	KIAA0864
24787	CCND2
24787	CCND2
28979	LMO2
15914	MAPK10
19346	SH3BP5
15864	MME
19238	LMO2
30263	ASB13
19291	MYBL1
19312	NEIL1
25036	FLJ12363
26385	MME
19227	LOC96597
22122	IRF4
16886	LRMP
24480	KIAA1039
27378	LRMP
27379	LRMP
24729	IRF4
27673	LRMP
19348	*Similar to
24429	BCL6
28472	MAPK10
26516	*Similar clone=417048
19268	BCL6 @Homo sapiH08 (LOC152137) Sur_clone=232
32529	2321
17646	BCL2

The resulting gene list that distinguishes ABC and GCB if the PAM analysis is performed only on the 31 best spots merged with the well known lymphoma genes. Marked in grey are the 31 best spots from all twelve thousand spots compared. Remarkably, the two classical lymphoma marker genes MAPK10 and CCND2 reach a similar quality in distinguishing ABC and GCB as the best separating ones.

**Table S8.** Cell cycle gene set that best distinguishes ABC and GCB subgroup. The genes are annotated by their spot ID, ensembl gene-ID and their gene name. Additionally the cell cycle states are given. The latter parameter shows a strong signal in the early and late cell cycle states compared with all available cell cycle states in the data set.

**SpotID**	**Ensembl ID**	**cell cycle state**	**Gene**
24927	ENSG00000165810	85	BTNL9
33929	ENSG00000165810	85	BTNL9
26913	ENSG00000138764	72	CCNG2
24750	ENSG00000136244	80	IL6
32430	ENSG00000162783	56	IER5
24491	ENSG00000165810	85	BTNL9
30172	ENSG00000138764	72	CCNG2
24930	ENSG00000187837	69	HIST1H1C
24725	ENSG00000011007	59	TCEB3
24908	ENSG00000118515	83	SGK
30355	ENSG00000164330	84	EBF
32096	ENSG00000164330	84	EBF
31931	ENSG00000164543	18	STK17A
26081	ENSG00000180447	80	GAS1
19374	ENSG00000124762	21	CDKN1A
24969	ENSG00000164330	84	EBF
24647	ENSG00000164330	84	EBF
34708	ENSG00000118515	83	SGK
27774	ENSG00000134058	92	CDK7
26401	ENSG00000118515	83	SGK
26725	ENSG00000164330	84	EBF
28881	ENSG00000163918	52	RFC4
17786	ENSG00000102804	1	TSC22D1
24613	ENSG00000102804	1	TSC22D1
33901	ENSG00000100644	2	HIF1A
27538	ENSG00000171656	96	ETV5
27952	ENSG00000179583	76	CIITA
34557	ENSG00000052841	2	TTC17
30021	ENSG00000099953	95	MMP11
27704	ENSG00000164330	84	EBF
26992	ENSG00000102804	1	TSC22D1
26344	ENSG00000138764	72	CCNG2
24832	ENSG00000163918	52	RFC4
26080	ENSG00000163739	76	CXCL1
33329	ENSG00000179583	76	CIITA
17290	ENSG00000134058	92	CDK7
30922	ENSG00000185658	5	BRWD1
26162	ENSG00000135541	91	AHI1
34288	ENSG00000134884	48	NA
33646	ENSG00000185658	5	BRWD1
26951	ENSG00000102804	1	TSC22D1
24977	ENSG00000153936	92	HS2ST1
16661	ENSG00000123080	75	CDKN2C
25942	ENSG00000145050	49	ARMET
22163	ENSG00000169926	6	KLF13
17405	ENSG00000178573	30	MAF
27275	ENSG00000100644	2	HIF1A
30415	ENSG00000164330	84	EBF
34484	ENSG00000151150	50	ANK3
33221	ENSG00000065809	2	FAM107B
32218	ENSG00000179583	76	CIITA
29637	ENSG00000145632	99	PLK2PLK2
27939	ENSG00000179583	76	CIITA
27328	ENSG00000108984	44	MAP2K6
28792	ENSG00000099326	53	ZNF42
30725	ENSG00000175455	65	CCDC14
16736	ENSG00000136244	80	IL6
30874	ENSG00000081320	77	STK17B
28707	ENSG00000123080	75	CDKN2C
33336	ENSG00000175455	65	CCDC14
15871	ENSG00000168310	7	IRF2
28640	ENSG00000100526	0	CDKN3
28748	ENSG00000136244	80	IL6
28430	ENSG00000168310	7	IRF2
26084	ENSG00000128590	38	DNAJB9
30859	ENSG00000117650	93	NEK2
28674	ENSG00000138061	66	CYP1B1
16127	ENSG00000138061	66	CYP1B1
24868	ENSG00000012963	52	C14orf130
30508	ENSG00000081320	77	STK17B
34108	ENSG00000169926	6	KLF13
16053	ENSG00000173757	83	STAT5B
16091	ENSG00000100526	0	CDKN3
33594	ENSG00000179583	76	CIITA
32924	ENSG00000185658	5	BRWD1
32766	ENSG00000135164	74	DMTF1
16597	ENSG00000109971	0	HSPA8

**Table S9.** The cell cycle genes, which were chosen to distinguish the ABC and the GCB group.

**Ensembl gene ID**	**cell cycle state**	**Gene symbol**
ENSG00000011007	59	TCEB3
ENSG00000012963	52	C14orf130
ENSG00000052841	2	TTC17
ENSG00000065809	2	FAM107B
ENSG00000081320	77	STK17B
ENSG00000099326	53	ZNF42
ENSG00000099953	95	MMP11
ENSG00000100526	0	CDKN3
ENSG00000100644	2	HIF1A
ENSG00000102804	1	TSC22D1
ENSG00000108984	44	MAP2K6
ENSG00000109971	0	HSPA8
ENSG00000117650	93	NEK2
ENSG00000118515	83	SGK
ENSG00000123080	75	CDKN2C
ENSG00000124762	21	CDKN1A
ENSG00000128590	38	DNAJB9
ENSG00000134058	92	CDK7
ENSG00000134884	48	NA
ENSG00000135164	74	DMTF1
ENSG00000135541	91	AHI1
ENSG00000136244	80	IL6
ENSG00000138061	66	CYP1B1
ENSG00000138764	72	CCNG2
ENSG00000145050	49	ARMET
ENSG00000145632	99	PLK2PLK2
ENSG00000151150	50	ANK3
ENSG00000153936	92	HS2ST1
ENSG00000162783	56	IER5
ENSG00000163739	76	CXCL1
ENSG00000163918	52	RFC4
ENSG00000164330	84	EBF
ENSG00000164543	18	STK17A
ENSG00000165810	85	BTNL9
ENSG00000168310	7	IRF2
ENSG00000169926	6	KLF13
ENSG00000171656	96	ETV5
ENSG00000173757	83	STAT5B
ENSG00000175455	65	CCDC14
ENSG00000178573	30	MAF
ENSG00000179583	76	CIITA
ENSG00000180447	80	GAS1
ENSG00000185658	5	BRWD1
ENSG00000187837	69	HIST1H1C

The cell cycle genes annotated by their ensembl gene-ID and their gene name. Additionally the cell cycle states are annotated. The latter parameter shows a strong signal in the early and late cell cycle states compared with all available cell cycle states in the data set.

**Table S10.** Gene expression values of the main regulatory network distinguishing ABC and GCB.

**Gene**	**ABC**	**GCB**
ASB13	−	+
MYBL1	−	+
MME	−	+
MAPK10	−	+
LRMP	−	+
LMO2	−	+
FN1	−	+
CTGF	−	+
COL3A1	−	+
BCL6	−	+
BCL7A	−	+
NEIL1	−	+
SH3BP5	+	−
BCL2	+	−
CCND2	+	−
IRF4	+	−
IGHM	+	−
FOXP1	+	−

Genes from Figure 2 and their gene expression values in the subgroups ABC and GCB are shown. The symbol “−” indicates a lower gene expression than “+”. In this network, more genes of the more aggressive ABC type have a lower gene expression than the GCB type.

**Table S11.** List of potential Notch target transcripts.

**Gene ID**	**Transcript ID**	**Description**
ENSG00000156136	ENST00000286648	Deoxycytidine kinase
ENSG00000148158	ENST00000277244	Sorting nexin family member 30
ENSG00000179388	ENST00000317216	Early growth response protein 3
ENSG00000198833	ENST00000361212	Ubiquitin-conjugating enzyme E2 J1
ENSG00000198833	ENST00000361333	Ubiquitin-conjugating enzyme E2 J1
ENSG00000065308	ENST00000182527	Translocation associated membrane protein 2
ENSG00000170584	ENST00000302764	NudC domain containing protein 2
ENSG00000074706	ENST00000265198	phosphoinositide-binding protein PIP3-E
ENSG00000134108	ENST00000256496	ADP-ribosylation factor-like 10C)

For all genes of the Lymphochip, all available transcripts annotated in ensembl were screened for the GY, Brd and K boxes. Only these transcripts bear all three boxes, GY, Brd and K in the 3′-UTRs. They are possible candidates to be regulated by the Notch signalling pathway. Moreover, the Deoxycytidine kinase (ENSG00000156136) and the Translocation associated membrane protein 2 (ENSG00000065308) show different gene expression values between the ABC and GCB subgroups.

**Table S12.** T-test result of network genes in another data set.

**Genes**	**P-value**	**T-value**
CCND2	6.260705e-06	5.56939706
BCL6	2.490035e-02	−2.34449786
BCL2	1.843571e-03	3.43618678
IRF4	2.082072e-07	6.49044833
LMO2	3.820841e-07	−6.66162303
MAPK10	3.888633e-02	−2.15403094

The genes from the proposed STRING-network in Figure 4 were used to apply a T-test between the ABC and the GCB group in the gene expression data of Shipp et al. The authors Wright et al. found some evidence for these DLBCL groups in there. The most obvious rejection of the null hypothesis is delivered by IRF4, LMO2, CCND2, BCL2, BCL6 and MAPK10, which are also part of the predictor of Wright et al.

## Figures and Tables

**Table 1.** Optimal molecular survival predictor applying six genes.

**Gene name**	**Gene description**
HLA-DPa	Major histocompatibility complex, class II, DP alpha 1
HLA-DQa	Major histocompatibility complex, class II, DQ alpha1
HLA-DRb5	Major histocompatibility complex, class II, DR beta 1
SEPT1	Serologically defined breast cancer antigen NY-BR-24=Similar to DIFF6
EIF2S2	Eukaryotic translation initiation factor 2 subunit 2
IDH3A	Isocitrate dehydrogenase 3 (NAD+) alpha

The gene symbol (left side) is followed by the gene description. Three of these genes are HLA major histocompatibility complex genes (HLA).

**Table 2.** Regulatory network of genes best distinguishing ABC and GCB.

**Functional categories**	**Gene**	**Description**
Proliferation	CCND2	cyclin D2, regulates G1 to S transition of CDK4/CDK6; CTGF, fibroblast growth factor
	MAPK10	map kinase 10
	MYBL1	transcriptional activator in the proliferation of neurons, spermatogenic and B-lymphoid cells (recognition sequence: 5′YAAC(GT)G-3′)
	ASB13	ankyrin repeat and sox box-containing protein 13, mediates protein-protein interactions, sox box couples suppressors of cytokine signalling and binding partners with elongin B and C complex to target them for degradation
	SH3BP5	SH3 domain binding protein, targets protein-protein interaction
Block of proliferation	MME	synonyms CALLA, common acute lymphocytic leukemia antigen, the synonym CD10 stresses its properties as a tumor suppressor gene
	BCL7A	putative tumor suppressor gene in T-cell lymphoma
Apoptosis	BCL2	integral outer mitochondrial protein to block apoptosis
	BCL6	transcriptional repressor, necessary for germinal center formation in lymph nodes
Differentiation	CTGF	fibroblast differentiation
	FOXP1	forkhead box P1
	LMO2	LIM domain only 2 transcription factor for hematopoetic development
	LAMP	expressed in lymphoid cells during development
	COL3A1	collagen type III
	FN1	fibronectin 1, cell adhesion
	NEIL1	base excision repair
Immune cell specific	IGHM	immunoglobulin heavy chain gene
	IRF4	interferon regulatory factor 4

The genes of the network in Figure 4 (suppl.) are associated to the functional categories “Proliferation”, “Block of proliferation”, “Apoptosis”, “Differentiation” and “Immune cell specific”, by their annotation. Most of them are part of the antagonists “Proliferation” and “Block of proliferation”. This indicates the complex regulation and importance of proliferation in the determination of ABC and GCB lymphomas. Classical lymphoma genes (see Table S4) known previously are given in *italics*.
